# Effect of shear stress and substrate on endothelial DAPK expression, caspase activity, and apoptosis

**DOI:** 10.1186/1756-0500-6-10

**Published:** 2013-01-10

**Authors:** Keith Rennier, Julie Y Ji

**Affiliations:** 1 Department of Biomedical Engineering, Indiana University Purdue University Indianapolis, 723 West Michigan Street, SL-220J, 46202, Indianapolis, IN, USA

**Keywords:** Shear stress, Substrate, DAPK, Apoptosis, TNFα

## Abstract

**Background:**

In the vasculature, misdirected apoptosis in endothelial cells leads to pathological conditions such as inflammation. Along with biochemical and molecular signals, the hemodynamic forces that the cells experience are also important regulators of endothelial functions such as proliferation and apoptosis. Laminar shear stress inhibits apoptosis induced by serum depletion, oxidative stress, and tumor necrosis factor α (TNFα). Death associated protein kinase (DAPK) is a positive regulator of TNFα induced apoptotic pathway. Here we investigate the effect of shear stress on DAPK in endothelial cells on glass or silicone membrane substrate. We have already shown a link between shear stress and DAPK expression and apoptosis in cells on glass. Here we transition our study to endothelial cells on non-glass substrates, such as flexible silicone membrane used for cyclic strain studies.

**Results:**

We modified the classic parallel plate flow chamber to accommodate silicone membrane as substrate for cells, and validated the chamber for cell viability in shear stress experiments. We found that adding shear stress significantly suppressed TNFα induced apoptosis in cells; while shearing cells alone also increased apoptosis on either substrate. We also found that shearing cells at 12 dynes/cm^2^ for 6 hours resulted in increased apoptosis on both substrates. This shear-induced apoptosis correlated with increased caspase 3/7 activities and DAPK expression and activation via dephosphorylation of serine 308.

**Conclusion:**

These data suggest that shear stress induced apoptosis in endothelial cells via increased DAPK expression and activation as well as caspase-3/7 activity. Most *in vitro* shear stress studies utilize the conventional parallel plate flow chamber where cells are cultured on glass, which is much stiffer than what cells encounter *in vivo*. Other mechanotransduction studies have utilized the flexible silicone membrane as substrate, for example, in cyclic stretch studies. Thus, this study bridges the gap between shear stress studies on cells plated on glass to studies on different stiffness of substrates or mechanical stimulation such as cyclic strain. We continue to explore the mechanotransduction role of DAPK in endothelial apoptosis, by using substrates of physiological stiffness for shear stress studies, and by using silicone substrate in cyclic stretch devices.

## Background

In this paper, we present an update to our previous publication where we began to analyze the role of death-associated protein kinase (DAPK) in apoptosis in sheared endothelial cells [1]. The endothelial lining of the blood vessel wall plays a critical role in the initiation and progression of cardiovascular diseases such as atherosclerosis which remain the leading cause of death in developed countries [2,3]. Endothelial cells exist in a mechanically active environment where they are continuously exposed to frictional wall shear stress and cyclic strain due to blood flow [4]. Along with biochemical and molecular signals, the hemodynamic forces that the cells experience on the lumen side are also important regulators of endothelial functions in cellular processes such as proliferation, apoptosis, migration, and wound healing [5,6]. In the vasculature, misdirected control of apoptosis in endothelial cells can lead to pathological conditions such as inflammation, clotting, and smooth muscle cells recruitment [7]. Increased cell turnover rate exists at sites prone to lesion development – areas of disturbed flow and low levels of shear stress [8,9]. On the other hand, laminar shear stress inhibits apoptosis induced by serum depletion, oxidative stress, and tumor necrosis factor (TNFα) [10-12].

Recent studies have revealed that the pro-apoptotic DAPK functions primarily as a mediator of programmed cell death [13]. Loss of DAPK expression in human carcinoma cell lines and its ubiquitous presence in many cell and tissues types suggest its importance in regulating apoptosis [14]. DAPK is activated following a variety of stimuli including TNFα, ceramide, interferon (IFN-γ), and oncogenes such as p53 [15-17], serving as a converging point for apoptotic signaling. DAPK is upstream of caspases, except caspase 8, and induces other caspase-independent cell death or autophagy [18]. DAPK is a 160 kDa, serine/threonine protein kinase that contains a calmodulin (CaM) binding domain, a cytoskeleton binding domain, eight ankyrin repeats, two P-loops which is a putative nuclear binding domain, plus an independent death domain necessary for promoting apoptosis [19]. Auto-phosphorylation of DAPK at Ser 308 is an important inhibitory regulatory checkpoint [20]. Serine 308 is in the Ca^2+^/CaM binding domain of DAPK, and is phosphorylated in growing cells. Apoptotic signals trigger dephosphorylation of serine 308, which along with CaM binding, are required for full activation of DAPK catalytic activities.

Precise mechanisms of DAPK functions in endothelial cells are largely unknown. Due to its location on the cytoskeleton, DAPK expression and activity are potentially susceptible to mechanical and structural stresses. We found that while cytokines such as TNFα induces apoptosis, adding shear stress significantly suppressed TNFα induced apoptosis; while shear stress alone also increased apoptosis. We also found that apoptosis was correlated with caspase activity and DAPK expression, suggesting that shear stress affected endothelial apoptosis by controlling DAPK expression. Like most *in vitro* shear stress studies, we used the conventional parallel plate flow chamber in our studies where endothelial cells were cultured on glass slides. Glass, which has elastic modulus on the order of 50 GPa, is much stiffer than what cells encounter *in vivo*, such as extracellular matrix (ECM), basement membrane, or other cells. In contrast, mechanotransduction studies that include cyclic stretch of endothelial and other cells have utilized fibronectin-coated silicone membrane as substrate, which has an average elasticity of 2 MPa [21-23]. We redesigned and constructed a flow chamber to accommodate cells plated on either glass or silicone membrane of similar size, in order to analyze the effect of different substrates on endothelial responses toward fluid shear stress. Our goal is to integrate mechanical cues such as shear stress and cyclic stretch, with mechanical properties of substrate, to fully understand the role of DAPK in apoptosis as part of endothelial mechanotransduction.

Here, we continue to investigate the effects of substrate on shear stress regulation of DAPK and apoptosis in bovine aortic endothelial cells (BAEC). We hypothesized that cells on the softer, flexible membrane substrate would be more protected from apoptosis under shear stress. We validated cell attachment and morphology on fibronectin-coated substrates, and found that shear stress induces apoptosis, through caspase and DAPK activation on both substrates. We also saw suppression of apoptosis subsequent to TNFα activation on membrane substrate, as we did on glass. Our data suggest that at least in terms of DAPK expression and apoptosis, endothelial cells respond in a similar way toward shear stress, independent of glass or silicone membrane substrates.

## Methods

### Cell culture and reagents

Bovine aortic endothelial cells (BAEC), passage number 4 to 10, were cultured in Dulbecco’s modified Eagle’s medium (DMEM) supplemented with 10% heat-inactivated fetal bovine serum (JR Scientific), 1% L-glutamine, and 2% penicillin streptomycin (all Sigma) and incubated at 37°C in humidified 5% CO_2_ environment. Human TNFα (Sigma) was reconstituted in water to a stock concentration of 10 μg/ml and added to static or shearing media for final working concentration of 10 or 25 ng/ml.

### Substrate preparation

Silicone sheets were obtained from Specialty Manufacturing. MTS testing done on the material in our laboratory obtained elastic modulus 1.2 to 1.5 MPa. Silicone membrane was cut to similar size as glass slides (38 × 75 mm), and placed in 10 cm petri dishes to be cleaned with ethanol, followed by sterilization under UV light. Both silicone and glass substrates were coated with 1.5 μg/ml fibronectin for at least 4 hours on an orbital shaker and stored at 4°C. BAEC at passage number 4 to 10 were passed and plated on 38 × 75 × 1 mm glass slides or membrane at approximately 500,000 cells per slide experiments and cultured for at least 24 hours to confluency before shear stress experiments.

### Shear stress

The parallel plate flow chamber was attached to a sterile, laminar flow system as previously described [24] in an environmental chamber kept at 37°C with 5% CO_2_. The magnitude of shear stress (*τ*) on the cell monolayer is calculated based on the Navier–Stokes equation for a Newtonian fluid in a parallel plate geometry. The equation for wall shear stress simplifies to: *τ* = 6*μQ*/(*bh*^2^), where *μ* is the viscosity of the media (0.01 dynes-sec/cm^2^), *Q* is the volumetric flow rate (~0.5 ml/s), *b* is the width of the flow chamber (2.5 cm), and *h* is the separation distance between the chamber and the glass slide (0.027 cm). The flow chamber is connected to a flow loop system where media is flown steadily from an upper to lower reservoir due to gravity, and the media is returned to upper reservoir by a peristaltic pump. The reservoirs act as buffers against any unsteadiness in flow. The rigid walls of the plastic flow chamber also prevent any significant stretch of the membrane substrate. Using this system, cells were exposed to 12 dynes/cm^2^ laminar wall shear stress. Flow experiments were done using regular growth media.

### Protein analysis

For protein analysis, cells were scraped off slides after each experiment and lysed with RIPA buffer with 0.5 mM PMSF, 150 mM protease inhibitor, 1 mM DTT, plus 50 μM sodium fluoride to preserve phosphorylated DAPK. Protein concentrations were measured using the colorimetric Bradford assay. Gel electrophoresis was done using NuPage 4-12% Bis-Tris SDS-PAGE gels (Invitrogen) loaded with equal sample protein amounts in each well, per manufacturer’s instruction. Gels were transferred to 0.45 μm nitrocellulose membrane (GE Technologies). After blocking for 1 hour, anti-DAPK 55 and anti-phospho-DAPK PS308 mouse antibodies (both Sigma) were used to detect protein expression at 1:1000 dilution, followed by goat anti-mouse HRP-conjugate secondary antibody (Bio-Rad) at 1:4000 dilution. Loading control was done using rabbit anti-actin antibody (Sigma) at 1:5000 dilution, followed by goat anti-rabbit HRP-conjugate secondary antibody (Bio-Rad) at 1:4000. Blocking and incubation with the anti-DAPK 55 antibody were done in 5% milk solution made with non-fat dry milk (Carnation) in PBS with 0.1% Tween (PBS-T) at room temperature. Blocking and incubation with the anti- anti-DAPK PS308 antibody were done in 5% milk solution in TBS with 0.1% Tween (TBS-T) to preserve phosphorylated antibody binding. Membranes were illuminated using Super Signal West Pico ECL reagents (Pierce). Imaging was done using BioRad Molecular Imager ChemiDoc XRS + System. Quantity One Image Analysis Software was used to quantitatively analyze band intensities.

### Caspase-Glo 3/7 apoptosis assay

Cells were plated on glass slides or silicon membranes at approximately 800,000 cells per slide 1 day prior to experiments. Sample sets were: glass control (static) and 6 hr shear, and membrane control (static) and 6 hr shear. Afterwards, cells were trypsinated and counted for each sample set, and adjusted to 15,000 cells per 50 μl of culture media. The Caspase assay was carried out as per manufacturer’s protocol. Briefly, using a 1:1 ratio of Caspase Glo® 3/7 Reagent (Promega) to cell culture media, cell suspensions and reagent were combined, gently mixed, and added to white 96-well plates. Wells containing media only were used as negative controls. After incubation for 1 hour at room temperature, luminescence of each well was read using a luminometer (PerkinElmer). Luminescence for each well was corrected for background readings, and normalized to control samples. Each sample was done in triplicates. All experimental samples were repeated at least 4 times (*n* = 4).

### TUNEL – flow cytometry

For TUNEL staining, cells were detached gently using non-enzymatic Cell Stripper (Mediatech) and collected for analysis. Cell suspensions were fixed with 1% parafomaldehyde and permeabilized with 0.2% Triton X-100, before TUNEL staining using In Situ Cell Death Detection Kit (Roche), according to manufacturer’s protocol. The FACScan flow cytometer (BD Biosciences) was used to categorize cells based on fluorescence characteristics, and Cell Quest Pro software (BD Biosciences) was used for data analysis. For the apoptosis results, all samples were replicated in least 4 different trials (*n* = 4).

### Statistics

All experiments were carried out for a minimum of three times, and each time samples were prepared and analyzed independently. Standard error of each group was calculated to verify the statistical significance of the results. Quantification of digital Western images was done to incorporate repeated blots for statistical analysis. Each Western band is corrected for background and the loading control on the same blot. Paired Student *t*-test was used to compare one condition between two sample sets [25,26]. *P* values less than 0.05 was considered sufficient for statistical significance.

## Results

### Shear stress significantly reduced apoptosis following TNFα activation on silicone substrate

**Figure 1:**
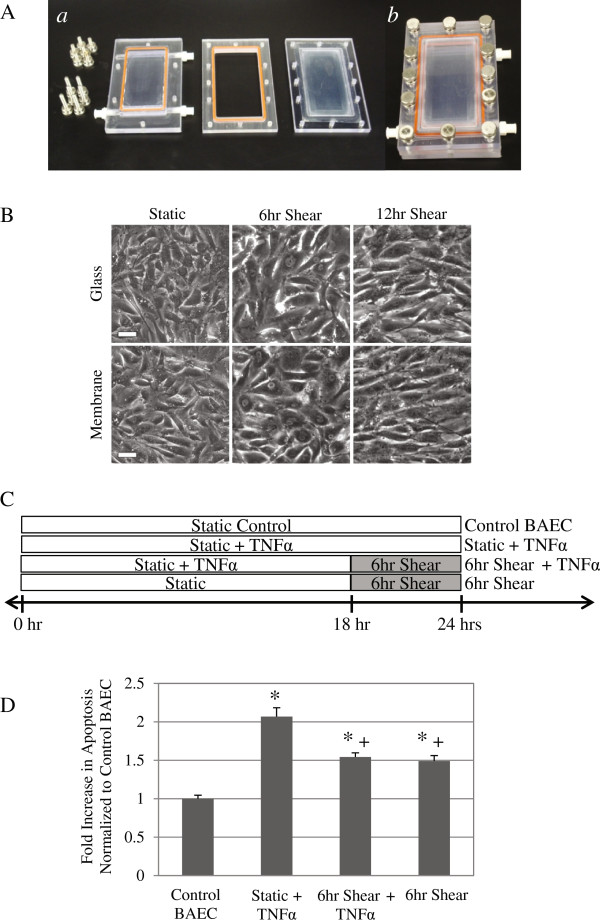
**Shear stress suppressed apoptosis subsequent to TNFα induction on cells plated on silicone membrane.** (**A**) Parallel plate design was redesigned to accommodate membrane substrate. (***a***) The top, middle, and bottom portion of parallel plate flow chamber, and (***b***) flow chamber assembled together. (**B**) BAEC spread on both glass and silicone membrane substrates, and after 24 hours cells reached confluency and formed uniform monolayer. Phase contrast images of static and sheared BAEC (12 dynes/cm^2^ for 6 and 12 hours) show that cells have begun to align with the direction of flow. White scale bar = 50 μm. (**C**) Diagram of 4 experimental groups. Control groups are static (Control BAEC) and 12 dynes/cm^2^ for 6 hours alone (6 hr Shear). Other cells were either incubated with TNFα (25 ng/ml) for a 24-hour period (Static + TNFα) or treated with TNFα for 18 hours followed by shearing at 12 dynes/cm^2^ for 6 hours (6 hr Shear + TNFα). We examined cell apoptosis using TUNEL staining. Results were quantified using FACS flow cytometry, and apoptosis data is presented as fold increase over control BAEC (**D**). TUNEL positive cells increased with TNFα treatment (25 ng/ml) (* *P* < 0.01), while subsequent shearing significantly reduced percentage of apoptotic cells (+ *P* < 0.05). Shear stress alone at 6 hours also promoted apoptosis, independent of TNFα treatment, though apoptosis was still significantly less than static TNFα treated cells. All data represent average ± standard error (*n* ≥ 5). * *P* < 0.01, compared to Control BAEC, + *P* < 0.05, compared to Static + TNFα.

We modified the current flow chamber to allow for use with either glass slide or a silicone membrane of similar size as substrate for cells (Figure 1A), and we have validated the new flow chamber with BAEC on glass slide or membrane, coated with fibronectin. Within 24 hours, cells have spread and reached a monolayer on either substrate, which can then be used for shear stress studies using our modified flow chamber. Experiments are conducted after cells reach confluency (Figure 1B). When cells have been sheared at 12 dynes/cm^2^ for over 6 hours, they begin to align in the direction of the flow on both substrates. Our data show that cells behave similarly on either glass or silicone substrates.

We have already shown that TNFα induces apoptosis in endothelial cells, and adding shear stress subsequent to TNFα treatment significantly suppresses apoptosis on glass [1]. We now confirm the same results for cells sheared on silicone membrane substrate. Cells were either incubated with TNFα (25 ng/ml) for a 24-hour period to fully induce apoptosis (Static + TNFα) or treated with TNFα for 18 hours followed by shearing at 12 dynes/cm^2^ in media containing TNFα for another 6 hours (6 hr Shear + TNFα). Control groups included static (Control BAEC) and cells sheared for 6 hours alone (6 hr Shear). The 4 experimental groups are depicted in Figure 1C. After TNFα and shear stimulation in flow chambers, apoptosis in endothelial cells was assessed by TUNEL staining with fluorescein label (Figure 1D). TNFα alone induced a significant two-fold increase in apoptotic cells compared to control BAEC on membrane (* *P* < 0.01). Applying shear stress (6 hrs) to cells already activated by TNFα (18 hrs) significantly decreased the fraction of apoptotic cells compared to non-sheared, TNFα treated cells (+ *P* < 0.05, compared to Static + TNFα). We also saw a significant increase of apoptosis in cells sheared for 6 hours compared to control BAEC (* *P* < 0.01); however, the result was still significantly less than TNFα treated static cells (+ *P* < 0.05). In fact, sheared BAEC, either with or without TNFα pretreatment, demonstrated similar levels of apoptosis that is significantly different from both static control and TNFα induction alone. These data correlates well with what we observed for cells on glass substrate, and suggests that while shear stress alone induces apoptosis, it also represses apoptosis subsequent to TNFα induction, regardless of substrate.

### Shear stress reduced DAPK expression following TNFα treatment on membrane substrates

**Figure 2:**
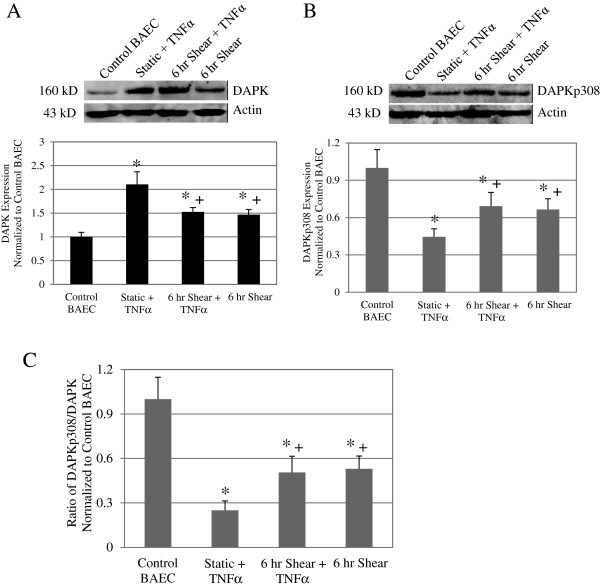
**Shear stress reduced DAPK expression following TNFα treatment on membrane substrates.** Western blot analysis for total DAPK expression (**A**) as well as phosphorylated serine 308DAPK (**B**) in Control BAEC, Static + TNFα, and 6 hr Shear + TNFα, and 6 hr Shear cells on membrane substrate. Each western blot image is representative of the DAPK or phospho-DAPK protein expression in each particular sample (Top panels). Loading control was assessed with anti-actin antibody on the same blot. We observed an increase total DAPK expression and a decrease of phospho-serine 308 DAPK after TNFα induction alone and in cells that were sheared for 6 h. Relative band intensity was quantified and analyzed based on results from 3 independent experiments, and presented as fold increase over control BAEC for overall DAPK and phospho-serine 308 DAPK (Bottom panels). Expression of total DAPK increased while phospho-serine 308 DAPK decreased following TNFα treatment compared to control BAEC. Adding shear stress to TNFα treatment or shearing cells alone significantly decreased DAPK expression compared to TNFα alone, but the difference was more significant in the phospho-DAPK analysis. Phosphorylated DAPK decreased to close to 50% with the addition of 6 h of shear stress, with or without prior TNFα treatment as compared to a near 26% decrease from TNFα treatment alone. (**C**) Phosphorylated DAPK as a fraction of total DAPK expression was calculated for each sample group based Western blots. All data represent average ± standard error (*n* = 3). * *P* < 0.01 compared to control cells, and + *P* < 0.05 compared to static TNFα treated cells.

As previously discussed, shear stress was shown to antagonize TNFα induced apoptosis on silicone membrane substrates. To further examine this apoptosis pathway, we wanted to investigate the subsequent shear stress effects on a key cell death mediator, DAPK, for cells plated on membrane substrates. For each membrane experiment, cells were treated as described above: incubated with TNFα (25 ng/ml) for a 24-hour period (Static + TNFα), treated with TNFα for 18 hours followed by shear for 6 hours (6 hr Shear + TNFα). Control groups were static (Control BAEC) or shear for 6 hours (6 hr Shear). Relative intensity of DAPK expression was quantified and normalized to the loading control, actin. Protein samples from at 3 separate experiments were analyzed. In Figure 2A, Western blot analysis showed a significant two-fold increase in DAPK expression in Static + TNFα. TNFα treated then sheared cells and cells that were sheared alone also showed 1.5-fold increase DAPK compared to Control BAEC (* *P* < 0.01). However, 6 hr Shear + TNFαcells showed a significant decrease in DAPK expression compared to cells treated with TNFα alone (*+ P* < 0.05). Representative blot and quantitative statistical analysis are presented, and these results reflect what we observed for cells on glass [1]. These results confirm that on membrane substrate TNFα and shear stress increase DAPK expression, and shear stress decreased DAPK expression after treatment with TNFα.

### Shear stress decreased DAPK activation in TNFα treated cells on membrane substrates

As an inhibitory checkpoint, DAPK is auto-phosphorylated in its static state. We analyzed phosphorylated DAPK expression (DAPKp308) in TNFα treated and sheared cells on membrane substrates. In Figure 2B, protein analysis showed significant decrease in phosphorylated DAPK at Ser 308 for Static + TNFα, 6 hr Shear + TNFα, and 6 hr Shear alone compared to Control BAEC (* *P* < 0.01). On the other hand, TNFα treated and then sheared cells displayed a significant increase in phospho-DAPK when compared to cells treated with TNFα alone (*+ P* < 0.05). Due to changes in both overall and phospho-DAPK, we evaluated the fraction of phospho-DAPK with respect to overall DAPK expression (Figure 2C). Western blot loading and transfer procedures were identical for both overall and phosphorylated DAPK protein analysis. Briefly, protein loading was standardized based on protein concentrations determined using Bradford protein assay. Each blot was incubated with antibody in 5% milk blotting solution at 1:1000 ratio. Therefore, the protein signal of DAPK or DAPKp308 is proportional to total DAPK or phosphorylated DAPK, respectively. The ratio of DAPKp308 to DAPK signal in turn, is proportional to percent of phosphorylated DAPK and demonstrates the overall DAPK activation in each sample group. Compared to Control BAEC, we see a significant decrease in ratio of phosphorylated DAPK, down to 25%, for TNFα treated cells. On the other hand, the ratio decreased to 50% and 53% respectively, for the 6 hr Shear + TNFα and 6 hr Shear cells (* *P* < 0.01). TNFα treated cells that were subsequently sheared displayed a smaller degree of DAPK activation, or higher Ser 308 phosphorylation, compared to TNFα treatment alone (*+ P* < 0.05). This data confirms that shear stress modulates DAPK expression and activation, possibly as another mechanism to antagonize TNFα induced apoptosis on membrane substrates, similar to cells on glass substrate.

### Shear stress alone induced apoptosis in cells on both glass and membrane substrates

**Figure 3:**
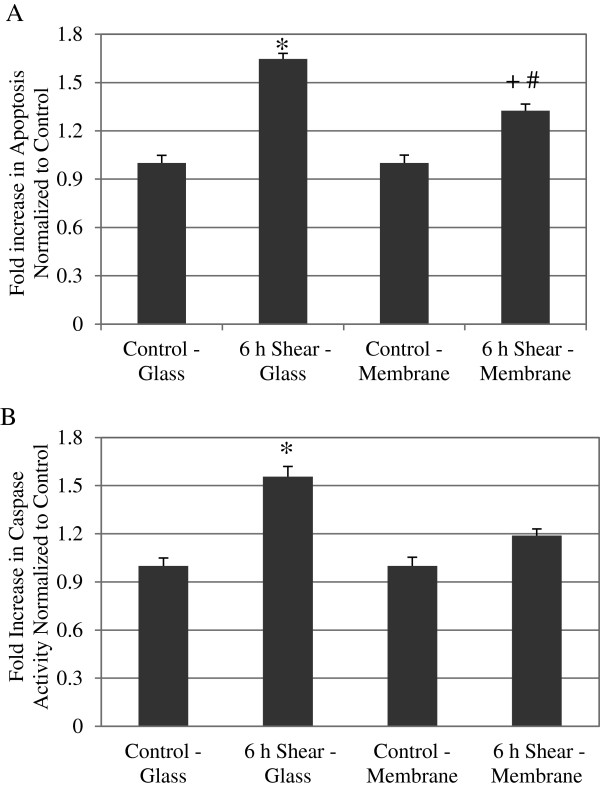
**Shear stress on cells plated on membrane induced apoptosis similar to cells on glass.** (**A**) TUNEL positive cells are presented as fold increase over static control on either substrate. Sheared cells demonstrated significant higher apoptosis compared to static control cells on both glass and membrane substrate. However, sheared cells on membrane cells exhibited significantly less apoptosis when compared to the sheared cell on glass. Data represent average ± standard error for *n* ≥ 4. * *P* < 0.01 compared to control-glass cells, + *P* < 0.01 compared to control-membrane cells, and # *P* < 0.01 compared to 6hr shear-glass. (**B**) Caspase-3 and −7 results are presented as fold increase over static control BAEC, and shear stress also induced significantly increased caspase 3/7 activities compared to static control cells on both substrates. Again, sheared cells on membrane exhibits significantly decreased caspase activity compared to sheared cells on glass. Data represent average ± standard error for *n* ≥ 5. * *P* < 0.01 compared to control-glass cells, + *P* < 0.05 compared to control-membrane cells, and # *P* < 0.05 compared to 6 hr shear-glass.

To this point, we have investigated the effect of shear stress on TNFα induced apoptosis for silicone membrane substrates. To further investigate the effect of shear stress alone for cells on glass or membrane substrate, we compared cellular apoptosis using TUNEL staining. We have previously shown that shear stress alone would increase apoptosis on glass slide [1]. Here cells plated on either glass or silicone membrane substrates were sheared at 12 dynes/cm^2^ for 6 hours, and after each experiment, cells were stained and quantified using flow cytometry. As shown in Figure 3A, there was a 1.64 fold increase in apoptosis for cells sheared on the glass substrate, when compared to control (* *P* < 0.01). For the membrane substrate, there was a 1.32 fold increase in apoptosis compared to control (+ *P* < 0.01). Furthermore, we also observed a significant decrease in apoptosis for cells sheared on membrane compared to glass substrate (# *P* < 0.01). There was no significant difference in apoptosis, however, between static control cells on either substrate. These data suggest that while shear stress induced apoptosis in cells on either substrate, cells on the softer membrane substrate demonstrate reduced apoptosis compared to glass.

### Shear-induced apoptosis correlated with increased caspase-3 and −7 activation

To analyze shear-induced endothelial apoptosis further, we examined caspase activity. Caspases are downstream of DAPK in the apoptotic pathway, and we had previously demonstrated increase of caspase 3/7 activity by shear stress alone in cells on glass [1]. Results from at least five different experiments were quantified for each case: control and sheared (12 dynes/cm^2^ for 6 hours) on glass or on membrane. Statistical analysis showed a significant increase of caspase 3/7 activity in sheared cells for both substrates when compared to control (Figure 3B). For cells on glass, we saw a 1.56 fold increase in caspase activity compared to control cells (* *P* < 0.01). Similarly for cells on the membrane, we saw a 1.2 fold increase compared to control (+ *P* < 0.05). This data suggest that shear induced apoptosis on glass and membrane was due to increased caspase 3/7 activities. Interestingly, we also observed a significant decrease in caspase 3/7 activities in cells sheared on membrane compared to glass (# *P* < 0.05); although, there was no statistical difference between control cells on the glass and membrane subsets. Our caspase-3/7 results showed a similar trend to what was observed in our quantitative analysis of the TUNEL results. This data suggests that shearing cells on silicone substrate, instead of glass, resulted in decreased caspase activities, and that shear-induced apoptosis in endothelial cells on both glass and membrane substrate correlated with increased caspase 3/7 activities.

### Shear stress increased overall DAPK expression cells on both glass and membrane substrates

**Figure 4:**
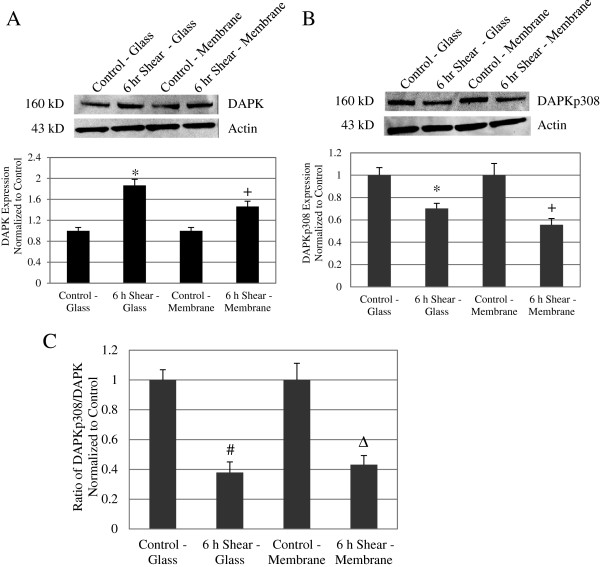
**Shear stress decreased DAPK phosphorylation at serine 308 on glass and membrane substrates.** (**A**) Western blot of overall DAPK expression in control and sheared cells (6 hours). Loading control bands are probed with anti-actin antibody on the same blot. DAPK expression increased with extended shear (Top). Quantitative analysis of band intensities was carried out on at least 4 separate sample sets. DAPK expression relative to actin is presented in the panel below as fold increase over control cells, for both glass and membrane samples. For both glass and membrane, DAPK expression increased with extended shear (Bottom). Data represent average ± standard error (*n* = 4) with * *P* < 0.01 compared to control-glass cells and + *P* < 0.05 compared to control-membrane cells. (**B**) Western blot of phosphorylated DAPK at serine 308, for control and sheared cells (6 hours). Loading control bands are probed with anti-actin antibody on the same blot (Top). Quantitative analysis of phospho-serine DAPK expression as fold increase over control, showed a decrease in phosphorylated DAPK serine 308 with 6hr shear, for both glass and membrane substrates (Bottom). Data represent average ± standard error (*n* = 4) with * *P* < 0.01 compared to control-glass cells and + *P* < 0.05 compared to control-membrane cells. (**C**) Phosphorylated DAPK as a fraction of total DAPK expression was calculated for each sample group based Western blot analysis. Compared to control cells, the fraction of phosphorylated DAPK decreased to a minimum of 38% for glass and 43% for membrane after 6 hours of shear stress. Data represent average ± standard error for 4 independent experiments (*n* = 4). # *P* < 0.01 compared to control-glass and Δ *P* < 0.01 compared to control-membrane.

Since the increase in apoptosis after 6 hours of shear stress alone correlated with increased caspase activity, we further anticipate DAPK to play a role in shear-induced apoptosis. Western blot analysis revealed that sheared cells (12 dynes/cm^2^, 6 hr)on glass and membrane substrate demonstrated a significant increase in DAPK expression compared to static control cells (Figure 4A Top). Relative intensity of DAPK expression was quantified and normalized to the loading control, actin. Protein samples from at least 4 separate experiments were analyzed, and we saw a 1.87 fold increase on glass and a 1.46 fold increase on membrane, in sheared cells compared to their respective control cells. The increase in DAPK in sheared cells on either substrate was significant: * *P* < 0.01 for cells on glass cells and # *P* < 0.05 for cells on membrane (Figure 4A Bottom). This result reflects what was previously reported for cells on glass substrate [1]. By comparing cells on membrane versus glass substrate, we found that there was no significant difference in DAPK expression in either the control or sheared cases. Our data suggests that, for cells on glass and membrane substrate, shear stress effectively increased DAPK expression after 6 hours.

### Shear stress decreased DAPK phosphorylation at serine 308 on glass and membrane substrates

DAPK is dephosphorylated at Ser 308 when it becomes activated. We analyzed phosphorylated DAPK expression (DAPKp308) in static and sheared cells on both glass and membrane substrates. Western blot analysis consistently showed a decrease in phosphorylated serine 308 DAPK in sheared cells (12 dynes/cm^2^, 6 hr) on both substrates when compared to their respective controls (Figure 4B Top). After quantifying repeated western blots, we found that sheared cells showed an average 30% decrease in DAPKp308 on glass (* *P* < 0.01) and 45% decrease on membrane (# *P* < 0.05), when normalized to static controls (Figure 4B Bottom). Again, there was no significant difference between substrates in either static or sheared case. Due to changes in both total and phosphorylated DAPK, we evaluated DAPKp308 as a fraction of total DAPK, which is an indication of overall DAPK activation, not just expression (Figure 4C). Both DAPK and DAPKp308 Western blot analysis were carried out as described above. Protein loading was normalized to actin, and the same Western blot procedure and analysis were carried out for both DAPK and DAPKp308. We saw that the ratio of phospho-DAPK decreased to 38% on glass and to 43% on membrane in sheared cells, as compared to their respective control cells (*P* < 0.01). These results are comparable to what was observed previously for cells on glass [1]. However, we saw no significant changes in Ser 308 phosphorylation between cells on glass and membrane in both control and sheared conditions. These data suggests that shear stress induced DAPK activation on membrane surfaces, similar to DAPK activation on glass surfaces. Expression and activation of DAPK by p308 dephosphorylation therefore are increased by fluid shear stress, regardless of substrate.

## Discussion

Understanding apoptosis, a fundamental cellular process, fully would have major impact on a multitude of cell and tissue types, as well as disease and therapeutic models. In atherosclerosis, for example, increased apoptosis contributes to prolonged inflammatory response, plaque instability, rupture, and thrombus formations [27]. Atherosclerosis develops preferentially at regions of disturbed flow and low shear stress, which are also sites of increased cell turnover rate [28], and shear stress has been shown to suppress cytokine induced apoptosis in endothelial cells [29]. DAPK is an important protein kinase in modulating apoptotic pathways [30]. Its potential role in endothelial apoptosis and mechanotransduction has been largely unexplored. Identifying the role of DAPK in endothelial cell function would be important to understanding apoptosis under both homeostatic and diseased conditions in the vasculature.

We have shown previously that DAPK is a mechano-sensitive regulator of apoptosis, in cells cultured on glass and sheared in parallel plate flow chambers [1]. Both apoptosis and expression and activation of DAPK are sensitive to fluid shear stress. Here we extend the study to cells cultured on a different substrate, silicone membrane, typically used for cyclic stretch studies. We again saw shear stress significantly attenuated TNFα activated apoptosis, and that shear stress alone also increased apoptosis (Figure 1D). Furthermore, we repeated our analysis of DAPK expression and activation in cells on membrane, and found that shear stress decreased TNFα activated DAPK expression and activation (Figure 2). This observation corresponds well to the results for cells on glass substrate. We continued to investigate further the effect of shear stress alone on cells plated on different substrates. We found that on either glass or membrane, shearing cells resulted in elevated apoptosis (Figure 3). Furthermore, shear-induced apoptosis on both substrates corresponded to an increase in caspase 3/7activities, and increased DAPK expression and activation via dephosphorylation of serine 308 (Figure 4). These data suggest that that in terms of apoptosis and DAPK expression, endothelial cells respond to shear stress similarly on either glass or silicone membrane substrate, and that DAPK expression in endothelial cells is more influenced by shear stress, rather than substrate.

Overall, regardless of substrate, we found that shear stress alone induced apoptosis, through a mechanism that involves increased DAPK expression and activation, upstream of caspase 3 and 7 activities. These findings agree well with our previous study, and further confirm that expression and activation of DAPK are regulated in part by fluid shear stress alone on both glass and membrane substrates. However, adding shear stress subsequent to TNFα induction suppressed both apoptosis and DAPK expression. This finding suggests that the anti-apoptotic effect of shear stress depends on the order of induction, in relation to pro-apoptotic agent. We are currently further investigating the competing effects of shear stress and apoptotic triggers such as TNFα.

When apoptosis or caspase results of Figure 3 were evaluated for statistical difference between substrates, instead of comparing static vs. shear, we did observe statistically significant differences between substrates. Both TUNEL and caspase-3 and 7 activities were significantly lower in cells sheared on membrane, compared to cells sheared on glass. There was no difference between the substrates under static condition. Furthermore, cells sheared on membrane substrate also demonstrated reduced DAPK expression and activation compared to cells sheared on glass, although the differences were not statistically significant (Figure 4). The reduced apoptosis and caspase activities in cells sheared on membrane suggest that while shear stress induced apoptosis overall, membrane substrate is more protective against apoptosis compared to glass substrate. The finding that shear-induced apoptosis was attenuated on membrane compared to glass suggests that there is at least some substrate-dependent differences in how endothelial cells respond to fluid shear stress.

Aside from its primary role in downstream activation in the death cascade, DAPK also regulates other cytoskeleton changes associated with apoptosis such as stress fibers and membrane blebbing [31]. DAPK also phosphorylates myosin regulatory light chain (MLC) at Ser19 [19,32], and promotes actomyosin contractility and stabilizes stress fibers in serum starved fibroblasts [32]. Thus, under shear, DAPK might be utilized for non-apoptotic functions such stress fiber formation, focal adhesion reorganization, and eventual re-alignment of cytoskeleton in the direction of flow [33]. Shear stress may induce structural changes in cells that are dependent on substrate. Other non-apoptotic functions of DAPK in endothelial cells would also be influenced by both shear stress and substrate.

In addition, mechanical properties of the substrate could also affect the substrate-dependent differences in apoptosis. Substrates stiffness is an important factor in behavior and functions of fibroblasts, smooth muscle cells, and neurons [34-36], and affects cell adhesion, shape, and cytoskeleton structure of endothelial cells [37]. Thus, substrate stiffness could be an important factor in determining how cells respond to external forces, and should be considered in conjunction with other mechanical cues such as shear stress. In addition to fluid shear stress, endothelial cells *in vivo* are also subject to cyclic stretch and reside on much softer substrate than glass. While most *in vitro* substrate studies have focused on cell behaviors under static conditions, other mechanotransduction studies have used cells cultured on glass surfaces for shear stress, or flexible silicone membrane for cyclic stretch devices.

This study bridges the gap between shear stress studies on cells plated on glass, to substrates of a more physiologically relevant stiffness. Our investigation on the mechanotransduction role of DAPK in endothelial apoptosis will continue in two fronts. We will examine the effect of shear stress on cells on substrates of varying mechanical properties, on the order of kPa. We plan to generate polyacrylamide hydrogels of different stiffness according to published protocols [38], which can be anchored to a base and accommodated into our flow chamber. We will also continue to use silicone membrane as substrate in a cyclic stretch device to examine the effect of substrate strain on endothelial apoptosis.

## Conclusions

In summary, we have shown that cells plated on either glass or silicone substrate showed increased apoptosis in response to fluid shear stress that also correlates with increased DAPK expression and activation, and caspase 3/7 activities. Cells are actively sensing and responding to their environment, which includes not only external and internal forces, but also mechanical properties of their surroundings [39]. This study provides the basis for continued research to further examine cellular apoptosis and DAPK activity in cells exposed to different substrate stiffness or to cyclic stretch. This study lays the groundwork for our goal to elucidate the simultaneous effect of multiple mechanical stresses (shear stress, cyclic stretch, and substrate stiffness) on endothelial functions such as apoptosis.

## Competing interests

The authors declare that they have no competing interests and no conflicts of interest.

## Authors’ contribution

KR carried out major experiments and data analysis of this study and helped draft and edit the manuscript with JYJ. JYJ contributed to the experimental design, data interpretation, editing, and submission of this manuscript. All authors have read and approved the final manuscript.

## References

[B1] Rennier K Ji JY Shear stress regulates expression of death-associated protein kinase in suppressing TNFalpha-induced endothelial apoptosis J Cell Physiol 2012 227 2398 2411 10.1002/jcp.22975 21826654

[B2] Teichert AM Scott JA Robb GB Zhou YQ Zhu SN Lem M Keightley A Steer BM Schuh AC Adamson SL Endothelial nitric oxide synthase gene expression during murine embryogenesis: commencement of expression in the embryo occurs with the establishment of a unidirectional circulatory system Circ Res 2008 103 24 33 10.1161/CIRCRESAHA.107.168567 18556578

[B3] Davies P Flow-mediated endothelial mechanotransduction Physiol Rev 1995 75 519 560 7624393 10.1152/physrev.1995.75.3.519 PMC3053532

[B4] Chien S Mechanotransduction and endothelial cell homeostasis: the wisdom of the cell Am J Physiol Heart Circ Physiol 2007 292 H1209 H1224 17098825 10.1152/ajpheart.01047.2006

[B5] Glagov S Zarins C Giddens D Ku D Hemodynamics and atherosclerosis. Insights and perspectives gained from studies of human arteries Arch Pathol Lab Med 1988 112 1018 1031 3052352

[B6] Davies PF Spaan JA Krams R Shear stress biology of the endothelium Ann Biomed Eng 2005 33 1714 1718 10.1007/s10439-005-8774-0 16389518

[B7] Choy JC Granville DJ Hunt DW McManus BM Endothelial cell apoptosis: biochemical characteristics and potential implications for atherosclerosis J Mol Cell Cardiol 2001 33 1673 1690 10.1006/jmcc.2001.1419 11549346

[B8] Caplan BA Schwartz CJ Increased endothelial cell turnover in areas of *in vivo* Evans Blue uptake in the pig aorta Atherosclerosis 1973 17 401 417 10.1016/0021-9150(73)90031-2 4123526

[B9] Ku D Giddens D Zarins C Glagov S Pulsatile flow and atherosclerosis in the human carotid bifurcation. Positive correlation between plaque location and low oscillating shear stress Arteriosclerosis 1985 5 293 302 10.1161/01.ATV.5.3.293 3994585

[B10] Dimmeler S Haendeler J Nehls M Zeiher AM Suppression of apoptosis by nitric oxide via inhibition of interleukin-1beta-converting enzyme (ICE)-like and cysteine protease protein (CPP)-32-like proteases J Exp Med 1997 185 601 607 10.1084/jem.185.4.601 9034139 PMC2196141

[B11] Dimmeler S Haendeler J Rippmann V Nehls M Zeiher A Shear stress inhibits apoptosis of human endothelial cells FEBS Lett 1996 399 71 74 10.1016/S0014-5793(96)01289-6 8980122

[B12] Hermann C Zeiher AM Dimmeler S Shear stress inhibits H2O2-induced apoptosis of human endothelial cells by modulation of the glutathione redox cycle and nitric oxide synthase Arterioscler Thromb Vasc Biol 1997 17 3588 3592 10.1161/01.ATV.17.12.3588 9437209

[B13] Cohen O Kimchi A DAP-kinase: from functional gene cloning to establishment of its role in apoptosis and cancer Cell Death Differ 2001 8 6 15 10.1038/sj.cdd.4400794 11313698

[B14] Michie AM McCaig AM Nakagawa R Vukovic M Death-associated protein kinase (DAPK) and signal transduction: regulation in cancer FEBS J 2009 277 74 80 19878310 10.1111/j.1742-4658.2009.07414.x

[B15] Cohen O Inbal B Kissil JL Raveh T Berissi H Spivak-Kroizaman T Feinstein E Kimchi A DAP-kinase participates in TNF-alpha- and Fas-induced apoptosis and its function requires the death domain J Cell Biol 1999 146 141 148 10402466 10.1083/jcb.146.1.141 PMC2199731

[B16] Chen RH Wang WJ Kuo JC The tumor suppressor DAP-kinase links cell adhesion and cytoskeleton reorganization to cell death regulation J Biomed Sci 2006 13 193 199 10.1007/s11373-005-9063-5 16456710

[B17] Pelled D Raveh T Riebeling C Fridkin M Berissi H Futerman AH Kimchi A Death-associated protein (DAP) kinase plays a central role in ceramide-induced apoptosis in cultured hippocampal neurons J Biol Chem 2002 277 1957 1961 10.1074/jbc.M104677200 11709549

[B18] Gozuacik D Bialik S Raveh T Mitou G Shohat G Sabanay H Mizushima N Yoshimori T Kimchi A DAP-kinase is a mediator of endoplasmic reticulum stress-induced caspase activation and autophagic cell death Cell Death Differ 2008 15 1875 1886 10.1038/cdd.2008.121 18806755

[B19] Kuo JC Lin JR Staddon JM Hosoya H Chen RH Uncoordinated regulation of stress fibers and focal adhesions by DAP kinase J Cell Sci 2003 116 4777 4790 10.1242/jcs.00794 14600263

[B20] Shohat G Spivak-Kroizman T Cohen O Bialik S Shani G Berrisi H Eisenstein M Kimchi A The pro-apoptotic function of death-associated protein kinase is controlled by a unique inhibitory autophosphorylation-based mechanism J Biol Chem 2001 276 47460 47467 10.1074/jbc.M105133200 11579085

[B21] Peng X Recchia FA Byrne BJ Wittstein IS Ziegelstein RC Kass DA *In vitro* system to study realistic pulsatile flow and stretch signaling in cultured vascular cells Am J Physiol Cell Physiol 2000 279 C797 C805 10942730 10.1152/ajpcell.2000.279.3.C797

[B22] Punchard MA Stenson-Cox C O’Cearbhaill ED Lyons E Gundy S Murphy L Pandit A McHugh PE Barron V Endothelial cell response to biomechanical forces under simulated vascular loading conditions J Biomech 2007 40 3146 3154 10.1016/j.jbiomech.2007.03.029 17561024

[B23] Lammerding J Fong LG Ji JY Reue K Stewart CL Young SG Lee RT Lamins A and C but not lamin B1 regulate nuclear mechanics J Biol Chem 2006 281 25768 25780 10.1074/jbc.M513511200 16825190

[B24] Frangos JA McIntire LV Eskin SG Shear stress induced stimulation of mammalian cell metabolism Biotechnol Bioeng 1988 32 1053 1060 10.1002/bit.260320812 18587822

[B25] Castets M Coissieux MM Delloye-Bourgeois C Bernard L Delcros JG Bernet A Laudet V Mehlen P Inhibition of endothelial cell apoptosis by netrin-1 during angiogenesis Dev Cell 2009 16 614 620 10.1016/j.devcel.2009.02.006 19386270

[B26] Harrison B Kraus M Burch L Stevens C Craig A Gordon-Weeks P Hupp TR DAPK-1 binding to a linear peptide motif in MAP1B stimulates autophagy and membrane blebbing J Biol Chem 2008 283 9999 10014 10.1074/jbc.M706040200 18195017

[B27] Martinet W Schrijvers DM De Meyer GR Thielemans J Knaapen MW Herman AG Kockx MM Gene expression profiling of apoptosis-related genes in human atherosclerosis: upregulation of death-associated protein kinase Arterioscler Thromb Vasc Biol 2002 22 2023 2029 10.1161/01.ATV.0000041843.44312.12 12482829

[B28] Berk BC Abe JI Min W Surapisitchat J Yan C Endothelial atheroprotective and anti-inflammatory mechanisms Ann N Y Acad Sci 2001 947 93 109 discussion 109–111 11795313 10.1111/j.1749-6632.2001.tb03932.x

[B29] Malek A Jiang L Lee I Sessa W Izumo S Alper S Induction of nitric oxide synthase mRNA by shear stress requires intracellular calcium and G-protein signals and is modulated by PI 3 kinase Biochem Biophys Res Commun 1999 254 231 242 10.1006/bbrc.1998.9921 9920763

[B30] Kimchi A DAP kinase and DAP-3: novel positive mediators of apoptosis Ann Rheum Dis 1999 58 Suppl 1 I14 I19 10577968 10.1136/ard.58.2008.i14 PMC1766573

[B31] Inbal B Bialik S Sabanay I Shani G Kimchi A DAP kinase and DRP-1 mediate membrane blebbing and the formation of autophagic vesicles during programmed cell death J Cell Biol 2002 157 455 468 10.1083/jcb.200109094 11980920 PMC2173279

[B32] Bialik S Bresnick AR Kimchi A DAP-kinase-mediated morphological changes are localization dependent and involve myosin-II phosphorylation Cell Death Differ 2004 11 631 644 15002035 10.1038/sj.cdd.4401386

[B33] Galbraith CG Skalak R Chien S Shear stress induces spatial reorganization of the endothelial cell cytoskeleton Cell Motil Cytoskeleton 1998 40 317 330 10.1002/(SICI)1097-0169(1998)40:4<317::AID-CM1>3.0.CO;2-8 9712262

[B34] Lo CM Wang HB Dembo M Wang YL Cell movement is guided by the rigidity of the substrate Biophys J 2000 79 144 152 10.1016/S0006-3495(00)76279-5 10866943 PMC1300921

[B35] Peyton SR Putnam AJ Extracellular matrix rigidity governs smooth muscle cell motility in a biphasic fashion J Cell Physiol 2005 204 198 209 10.1002/jcp.20274 15669099

[B36] Flanagan LA Ju YE Marg B Osterfield M Janmey PA Neurite branching on deformable substrates Neuroreport 2002 13 2411 2415 10.1097/00001756-200212200-00007 12499839 PMC2408859

[B37] Yeung T Georges PC Flanagan LA Marg B Ortiz M Funaki M Zahir N Ming W Weaver V Janmey PA Effects of substrate stiffness on cell morphology, cytoskeletal structure, and adhesion Cell Motil Cytoskeleton 2005 60 24 34 10.1002/cm.20041 15573414

[B38] Tse JR Engler AJ Preparation of hydrogel substrates with tunable mechanical properties Curr Protoc Cell Biol 2010 47 1 16 10.1002/0471143030.cb1016s47 20521229

[B39] Discher DE Janmey P Wang YL Tissue cells feel and respond to the stiffness of their substrate Science 2005 310 1139 1143 10.1126/science.1116995 16293750

